# Impact of systemic antimicrobial therapy on the faecal microbiome in symptomatic dairy cows

**DOI:** 10.1371/journal.pone.0296290

**Published:** 2024-01-05

**Authors:** Rose M. Collis, Patrick J. Biggs, Sara A. Burgess, Anne C. Midwinter, Gale Brightwell, Adrian L. Cookson

**Affiliations:** 1 AgResearch Ltd, Hopkirk Research Institute, Massey University, Palmerston North, New Zealand; 2 Molecular Epidemiology and Public Health Laboratory, School of Veterinary Science, Massey University, Palmerston North, New Zealand; 3 School of Natural Sciences, Massey University, Palmerston North, New Zealand; 4 New Zealand Food Safety Science and Research Centre, Massey University, Palmerston North, New Zealand; University of Nicolaus Copernicus in Torun, POLAND

## Abstract

Antimicrobial resistance is a global threat to human and animal health, with the misuse and overuse of antimicrobials suggested as the main drivers of resistance. Antimicrobial therapy can alter the bacterial community composition and the faecal resistome in cattle. Little is known about the impact of systemic antimicrobial therapy on the faecal microbiome in dairy cows in the presence of disease. Therefore, this study aimed to assess the impact of systemic antimicrobial therapy on the faecal microbiome in dairy cows in the pastoral farm environment, by analysing faecal samples from cattle impacted by several different clinically-defined conditions and corresponding antimicrobial treatments. Analysis at the individual animal level showed a decrease in bacterial diversity and richness during antimicrobial treatment but, in many cases, the microbiome diversity recovered post-treatment when the cow re-entered the milking herd. Perturbations in the microbiome composition and the ability of the microbiome to recover were specific at the individual animal level, highlighting that the animal is the main driver of variation. Other factors such as disease severity, the type and duration of antimicrobial treatment and changes in environmental factors may also impact the bovine faecal microbiome. AmpC-producing *Escherichia coli* were isolated from faeces collected during and post-treatment with ceftiofur from one cow while no third-generation cephalosporin resistant *E*. *coli* were isolated from the untreated cow samples. This isolation of genetically similar plasmid-mediated AmpC-producing *E*. *coli* has implications for the development and dissemination of antibiotic resistant bacteria and supports the reduction in the use of critically important antimicrobials.

## Introduction

Antimicrobial resistance (AMR) is a growing public and animal health issue [1, 2] and the use of antimicrobials in food-producing animals has become an established consumer concern [3]. Antimicrobial use, either for disease treatment or prophylactically, can alter both the bacterial community composition and resistome in cattle, swine, and chicken faeces [4–6]. Such perturbations in the microbiome can either be transient where the bacterial composition recovers, or microbiome changes may be long-lasting [7, 8]. Broad-spectrum antibiotics generally reduce the bacterial microbiome diversity [8, 9], however, the recovery and resilience of the microbiome can differ depending on the spectrum of the antimicrobial used and other host-dependent factors [10] such as diet [11]. The ecological balance of the bovine faecal microbiome is complex and can be affected by factors other than antimicrobial therapy such as feed type and concentrate use [12, 13], gastrointestinal disease [14], as well as farm management factors such as housing (e.g. indoor barns) [15].

There is growing concern that antimicrobial use in food-producing animals may increase the prevalence of antimicrobial resistant bacteria and antimicrobial resistance genes [3, 16], and increase the likelihood for horizontal gene transfer of such genes to pathogenic bacteria. The New Zealand Veterinary Association (NZVA) has an antimicrobial traffic light tier system that aims to increase antimicrobial stewardship, promote judicious use, and limit the potential for selection of key antibiotic resistance mechanisms [17].

To understand the impact of antimicrobial therapy on the faecal resistome in cattle, it is important to consider the microbiome composition and diversity which may influence resistome structure. Most studies conducted in food-producing animals, such as cattle, have evaluated the use of specific antimicrobial treatments or feed additives in controlled environments or experiments, often in the absence of clinical disease, however further work is required to understand the recovery of the microbiome post-treatment with systemic antimicrobials. Therefore, the aim of this study was to assess the impact of systemic antimicrobial therapy on the faecal microbiome of sick dairy cattle on working NZ dairy farms, by analysing faecal samples from cattle affected by several clinically-defined conditions and corresponding antimicrobial treatments.

## Materials and methods

### Study population and sample collection

Dairy cows from No. 1 Dairy Farm (Dairy 1) and No. 4 Dairy Farm (Dairy 4) research farms were recruited in the study on an opportunistic basis at the farm manager’s discretion upon presentation with clinical disease and subsequent veterinarian advice to treat with systemic antibiotics. The dairy farms in Palmerston North, New Zealand are <5 km apart and both operate a closed dairy farm system (animals are not introduced into the herd). The two farms are pasture-based, with the use of supplementary feed when required, and have a spring calving system. Associated faecal samples were collected over 14 months (1 July 2019–30 September 2020), however, samples were not collected between 23 March to 13 May 2020 due to the Covid-19 pandemic restrictions in NZ. This research was approved by the Massey University Animal Ethics Committee (protocol number 18/123).

**Fig 1:**
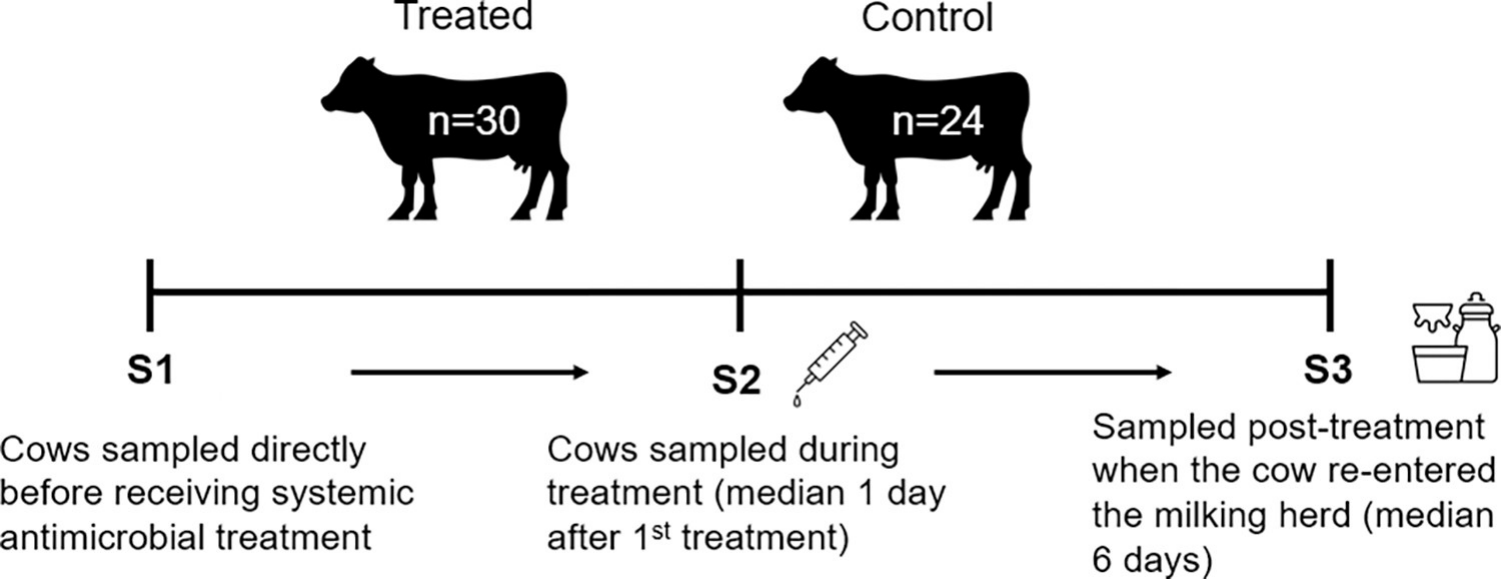
Timeline for faecal sampling. Experimental sampling days are indicated on the figure (S1, S2 and S3) and the number of treated and control (untreated) cows is shown at the top of the figure. The cows re-entered the milking herd at the timepoint associated with sample S3. Healthy control cow samples were collected at the same time as those of the treated cows.

Faecal samples were collected from cows receiving systemic antimicrobial therapy pre- (S1), during- (S2) and post-treatment (S3) (Fig 1). S1 samples were collected before the cow received antimicrobial treatment, sample S2 was collected during antibiotic treatment and was dependent on the prescribed treatment course (median one day after first treatment) and sample S3 was collected post-treatment once the recovered cow re-entered the milking herd (median six days). Samples from healthy cows were collected in the same sampling period as an untreated control to account for temporal variation in farm management factors such as feed type, as well as any farm-specific variation throughout the duration of the experiment. The control animals were selected on an *ad hoc* basis by farm staff and were from the same farm as treated animals. Cows that had received systemic antimicrobial therapy within the six months prior to sample collection were not eligible as controls. Where possible controls were matched with more than one treated cow if the treatment was started on the same day, hence the differing numbers between groups. Three dairy cows underwent caesarean surgery for veterinary teaching purposes and received ceftiofur as a broad-spectrum antibiotic during the procedure as per veterinary professional recommendations.

Each treated cow was assigned a number (1–30) and the respective control was designated using the same numbering system (1C - 30C). Faecal sample collection was performed by the farm staff. Briefly, dairy cows were safely restrained and approximately 5 g of faeces was collected and stored in a sterile pottle (the first portion was discarded to avoid potential contamination). Samples were stored at 4°C and processed within 24 hours of collection. A questionnaire regarding animal health, prescribed treatment course, and antimicrobial treatment history was completed by farm staff.

### Sample processing

Faecal samples were homogenised and 0.2 g was stored in triplicate at -80°C until DNA extraction. Faecal samples were enriched in phosphate-buffered peptone water (BD Difco^TM^, Fort Richard Laboratories, Auckland, New Zealand) at 35°C for 18 hours. After incubation 1 mL of the enrichment was mixed with glycerol (30%[v/v]) and stored at -80°C. In addition, 1 mL of the enrichment was pelleted at 17,000 x g and the pellet washed twice with phosphate-buffered saline (10 mM, pH7.3). The pellet was re-suspended in 1 mL molecular biology grade water and heated at 100°C for 10 min. These boiled lysate preparations were used as DNA template for PCR reactions.

### 16S rRNA gene V3-V4 sequencing

#### DNA extraction and sequencing

Faecal samples were defrosted on ice and transferred to a bead-beating tube. Genomic DNA was extracted from 0.2g faeces using the Presto Stool DNA Extraction Kit (Geneaid Biotech Ltd, New Taipei City, Taiwan) according to the manufacturer’s instructions with minor modifications. Briefly, for lysis the sample was vortexed at maximum speed for 7 min and a RNAse treatment step was included by adding 5 μL RNAse A (100 mg/mL, QIAGEN, Hilden, Germany) and incubating for 10 min at 37°C. To elute the DNA, 30 μL of elution buffer was added to the centre of the GD column and left to stand for 2 min. The sample was centrifuged at 16,000 x g for 2 min and the elution step repeated using the eluted DNA. The DNA concentration was quantified using a Qubit 4.0 fluorometer (ThermoFisher Scientific Inc., USA) and the A_260/280_ and A_260/230_ ratios were determined using a Nanodrop microvolume spectrophotometer (Nanodrop 2000c, ThermoFisher Scientific Inc., USA). DNA integrity and size was visualised on a 0.8% [w/v] agarose gel (UltraPure Agarose, Invitrogen, USA) stained with RedSafe (iNtRON Biotechnology, South Korea) using a high molecular weight *Hin*dIII/λ ladder (ThermoFisher Scientific Inc., USA).

Sequencing was performed using an Illumina MiSeq v2 platform with 2 x 250 base paired-end sequencing reads (Massey Genome Service, Massey University, Palmerston North, New Zealand) using dual index primers which flank the V3-V4 hypervariable region of the 16S rRNA gene (16SF V3 and 16SR V4 primers, as detailed in [18]).

### Bioinformatics

Adapter sequences and PhiX control library reads were removed from the demultiplexed fastq sequencing reads using FASTQ-MCF [19] and the read quality was assessed to a Phred score of Q20 by the Massey Genome Service. The data was analysed using the dada2 package v1.16.0 [20] in R v4.0.2 [21]. The demultiplexed reads were quality filtered and 10 bp and 40 bp trimmed from the forward and reverse reads, respectively. The denoised reads were merged, the chimaeras removed and the reads remaining post-processing were used to construct an amplicon sequence variant (ASV) table. Taxonomy was assigned using the SILVA v138.1 database which is formatted for dada2 [22–25]. A phyloseq object was constructed using phyloseq v1.32.0 [26] and diversity metrics were calculated using vegan v2.6.4 [27]. Pairwise comparison of the α-diversity of the faecal samples for health status and sample order was compared using ggpubr v0.4.0 [28] using the Wilcoxon test. The data was normalised to the median sequencing depth prior to calculating the β-diversity indices.

For the ceftiofur case study, as the three cows were undergoing treatment for the same procedure (caesarean), differential abundance analysis was performed using edgeR [29] by comparing pre- (S1), during (S2) and post-treatment (S3) faecal samples between treated (n = 3) and control (n = 3) cows, as well as within each treatment group. ASVs which were detected >1 times across 25% of the samples were included in the differential abundance analysis and the taxa were agglomerated at the genus level. Individual animals within treatment groups were considered as replicates. The differential abundance was determined by a generalised linear model implemented in edgeR [29] and p-values were adjusted for multiple comparisons using the Benjamini-Hochberg method to control the false discovery rate. Data visualisation was conducted in R using ggplot2 v3.3.5 [30], tidyverse v1.3.0 [31], dplyr v1.0.5 [32] and the website Colorgorical [33].

### Molecular characterisation and microbiological methods

The Qiagen DNeasy PowerSoil Pro Kit (QIAGEN, Hilden, Germany) was used to extract DNA from the faecal samples from animals in the ceftiofur case study and the DNA was used as input in subsequent PCR reactions to detect plasmid-mediated AmpC (pAmpC) gene families [34]. To isolate third-generation cephalosporin resistant *E*. *coli*, faecal enrichments from the ceftiofur treated cows (n = 3) and associated control cows (n = 3) were plated on to MacConkey agar (Fort Richard Laboratories, Auckland, New Zealand), MacConkey agar supplemented with 1 μg/mL cefotaxime sodium (Sigma-Aldrich, St. Louis, MO, USA), MacConkey agar supplemented with 1 μg/mL ceftazidime pentahydrate (Sigma-Aldrich, St. Louis, MO, USA) and CHROMagar™ ESBL (Fort Richard Laboratories, Auckland, New Zealand).

Presumptive *E*. *coli* were identified by matrix-assisted laser desorption/ionization-time of flight (MALDI-TOF) mass spectrometry (MS) (Bruker Daltonics, Billerica, CA, USA) using the “on slide formic acid extraction” method [35]. Kirby-Bauer disc diffusion antimicrobial susceptibility testing was undertaken on *E*. *coli* isolates for cefotaxime (30 μg), cefoxitin (30 μg), cefpodoxime (10 μg), tetracycline (30 μg), streptomycin (10 μg) and ciprofloxacin (5 μg) (Mast Group Ltd., Liverpool, United Kingdom) according to Clinical and Laboratory Standards Institute (CLSI) guidelines [36]. Presumptive AmpC and ESBL phenotypes were confirmed using either a three-disc (D69C AmpC disc test, Mast Group Ltd., Liverpool, United Kingdom) or double-disc comparison assay (D62C cefotaxime and D64C ceftazidime ESBL disc tests, Mast Group Ltd., Liverpool, United Kingdom), respectively. *E*. *coli* with an AmpC phenotype were tested for pAmpC gene families using a multiplex PCR [34] and isolates positive for the CITM primer set (targeting LAT-1 to LAT-4, CMY-2 to CMY-7 and BIL-1) [34] were subsequently tested using the CMY-2 primers [37], indicative of specific types of CMY-positive *E*. *coli*.

### Whole genome sequencing

#### DNA extraction

Three *E*. *coli* were selected for whole genome sequencing according to sample metadata (animal and sample order) and antimicrobial susceptibility testing results. Bacterial isolates from glycerol broths were inoculated on Columbia Sheep Blood agar (5% blood) (Fort Richard Laboratories, Auckland, New Zealand) and incubated for 18 hours at 35°C. An individual colony was subsequently sub-cultured under the same growth conditions to ensure purity. Cultures were sent on LB-agar slopes to the Institute of Environmental Science and Research (Christchurch, New Zealand) where the DNA was extracted with the Qiagen DNeasy Blood and Tissue kit (QIAGEN, Hilden, Germany) using a QIAcube (QIAGEN, Hilden, Germany). The DNA libraries were prepared using the Nextera XT v2 Library Preparation Kit (Illumina, San Diego, CA, USA) and sequenced on a NextSeq 550 sequencer (Illumina, San Diego, CA, USA) by the Institute of Environmental Science and Research (Christchurch, New Zealand).

### Genome analysis

Raw sequencing reads were processed using the Nullarbor v2.0 [38] pipeline. Briefly, the adapters were removed using Trimmomatic v0.39 [39], the Kraken v1.1.1 database [40] was used for species identification, genomes were assembled with SKESA v2.4.0 [41], and annotated with Prokka v1.14.6 [42]. Sequence typing was determined using mlst v2.19.0 [43] and PubMLST [44], the resistome profiles were assessed with ABRicate v1.0.1 [45] using the ResFinder database (downloaded 19/04/2020) [46–48], and the Centre for Genomic Epidemiology website [49] was used to detect the virulence genes and serotype in assembled genomes using the VirulenceFinder 2.0.3 database which contains 139 genes(v2020-05-29) [48, 50, 51] and the SerotypeFinder 2.0.1 database (v1.0.0) [52], respectively. MOB-suite [53, 54] was used to reconstruct and type plasmids from the draft genome assemblies. Antimicrobial resistance and virulence genes were identified in the plasmid sequences using ResFinder (v2022-08-08) [46–48] and VirulenceFinder (2022-12-02) [48, 50, 51], respectively. The single nucleotide polymorphism (SNP) variation was assessed using Snippy v4.4.3 [55] with isolate DF0049.2e (a ST57 CMY-2 producing *E*. *coli* isolated previously from Dairy 1 [56]) included as the reference genome.

## Results

Microbiome analysis of faecal samples from 30 treated and 24 healthy (untreated) cows was performed (Fig 1; S1 Table). Samples were collected pre-treatment (S1), during treatment (S2) (median one day after first treatment), and post-treatment (S3) (median six days after first treatment). For one control cow, only two faecal samples were collected. Therefore, 161 faecal samples were analysed and from these, 15263 unique 16S rRNA ASVs were observed. Of these, 118 were singletons and 1687 were doubletons, highlighting the high diversity and variation of ASVs within the faecal samples. The minimum, maximum, and mean number of reads across the 161 faecal samples was 16100, 88841 and 48002 reads, respectively.

### Microbiome diversity

**Fig 2:**
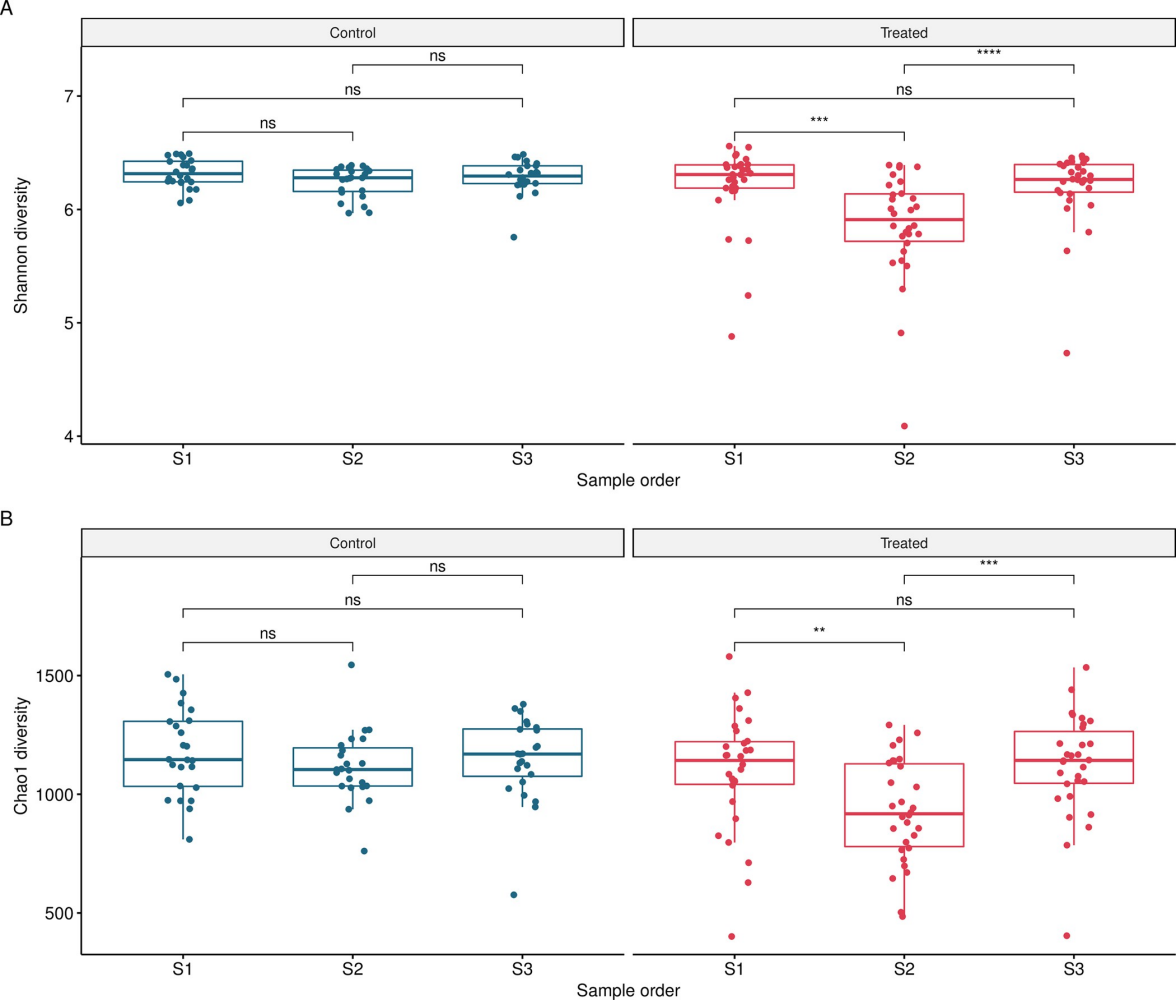
Diversity of faecal samples from treated (n = 30) and control (n = 24) cows. Diversity of faecal samples from treated (n = 30) and control (n = 24) cows for **A**: Shannon diversity and **B**: Chao1 diversity. Pairwise comparison of the α-diversity of the faecal samples were compared for health status and sample order; **, p<0.01; ***, p<0.001; ****, p<0.0001; ns, not significant, p>0.05.

A comparison of the Shannon diversity from the faecal microbiomes of treated and untreated cows suggested that the microbiota was diverse and species-rich (Fig 2A). For the treated cows, the Shannon diversity decreased during treatment (S2) compared to pre- (S1; p<0.0001) and post-treatment (S3; p<0.0001) samples, indicating that the microbiome composition of these samples was less diverse with fewer abundant ASVs. The Shannon diversity from the treated cow faecal samples collected post-treatment (S3) was higher compared to S2 (during) and was more comparable with pre-treatment (S1) samples (Fig 2A). No statistically significant changes in α-diversity for control cows were observed (samples S1, S2 and S3). The Chao1 diversity index indicated that the community richness was relatively even across the samples (Fig 2B), suggesting a similar number of ASVs in the faecal microbiomes, except from during treatment samples (S2) for treated cows which had a lower Chao1 diversity index (S1, p<0.005; S3, p<0.001).

**Fig 3:**
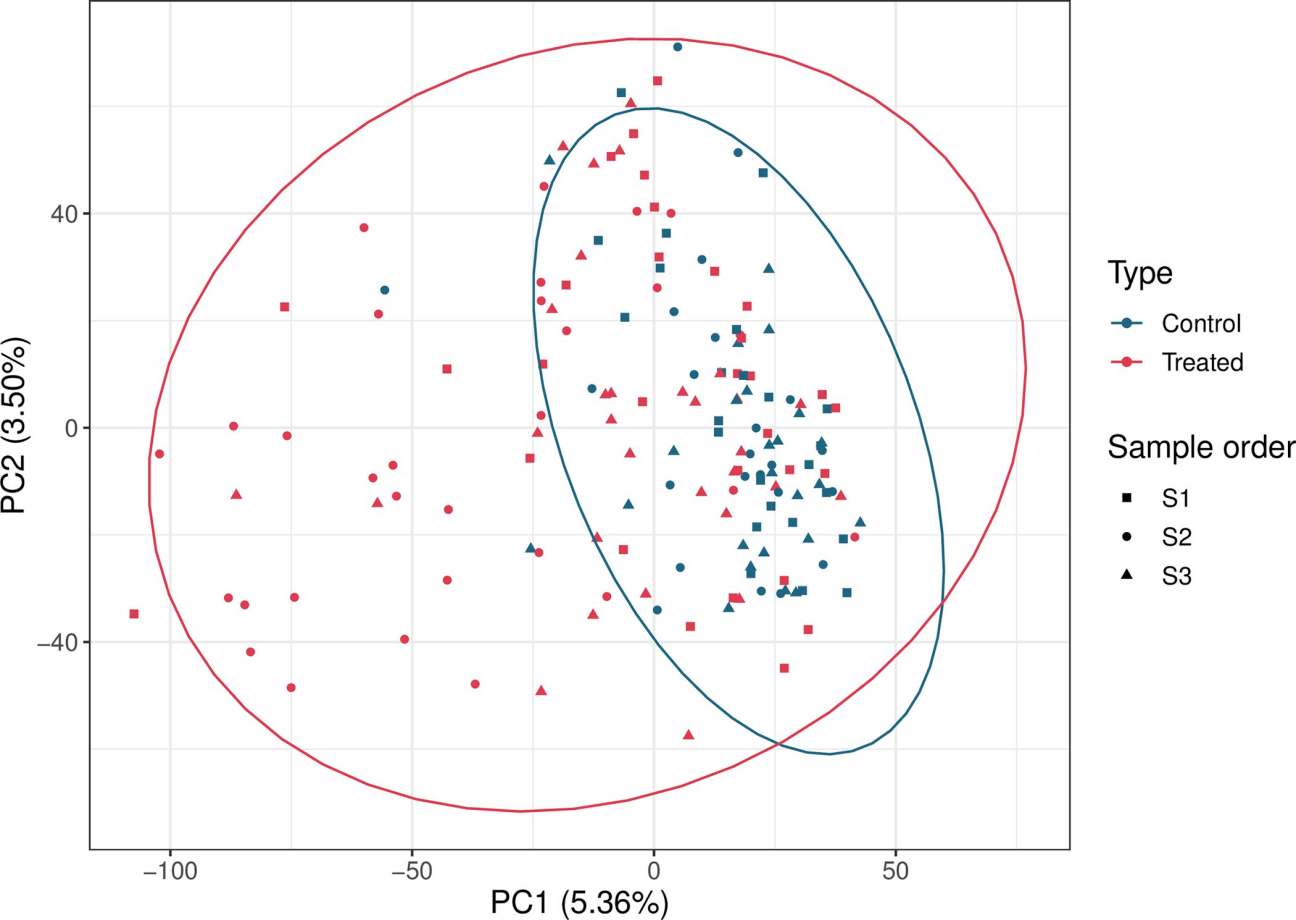
Principal component analysis for the faecal microbiome in treated and control cows. Principal Component Analysis for the faecal microbiome in treated animals (red) and control animals (blue) over time (shapes). The percentage of variation explained in the Principal Component Analysis is indicated on the axis labels.

The microbiome profiles did not cluster according to health status or sample order (S1, S2 and S3), with a high degree of between-sample and animal variation indicated through Principal Component Analysis (PCA) (Fig 3, PC1: 5.36%, PC2: 3.50%). The faecal microbiomes from treated cows were more dispersed compared to the microbiomes from control cows. Although the S2 faecal microbiomes from treated cows did not cluster together, they were generally more dispersed compared to the S1 and S3 samples. The dispersion of the faecal microbiome samples in the PCA plot and the low variance explained may be attributed to the variety of factors that can influence the microbiome composition such as variance at the individual animal level as well as treatment with specific antimicrobials and clinical diseases. Therefore, the faecal microbiomes from individual cows were further analysed as case studies differentiated by antimicrobial treatment type.

### Case studies

The animals were grouped and analysed by antimicrobial treatment, which included procaine penicillin G (n = 23 cows), ceftiofur (n = 3 cows), penethamate hydriodide (n = 3 cows), and a combination of marbofloxacin/penethamate hydriodide (n = 1 cow). Procaine penicillin G and penethamate hydriodide are classified as green tier by the NZVA, whereas marbofloxacin and ceftiofur are classified as red tier [17], with their use recommended only for the treatment of specific organisms or resistant infections. The untreated control cows associated with each treated cow were analysed in the respective case study.

### Procaine penicillin G treated cows

There was a high level of variation in the microbiome composition of the 23 cows treated with procaine penicillin G (S1 Fig) including at the individual animal level. To explore microbiome changes in more detail, four treated cows with contrasting clinical disease types, and their associated controls were analysed as a case study (i) cow 9: treated for between claw/foot rot, (ii) cow 10: treated for left displacement of the abomasum, (iii) cow 16: treated for a swollen vulva and (iv) cow 24: treated for a retained foetal membrane post-calving.

**Fig 4:**
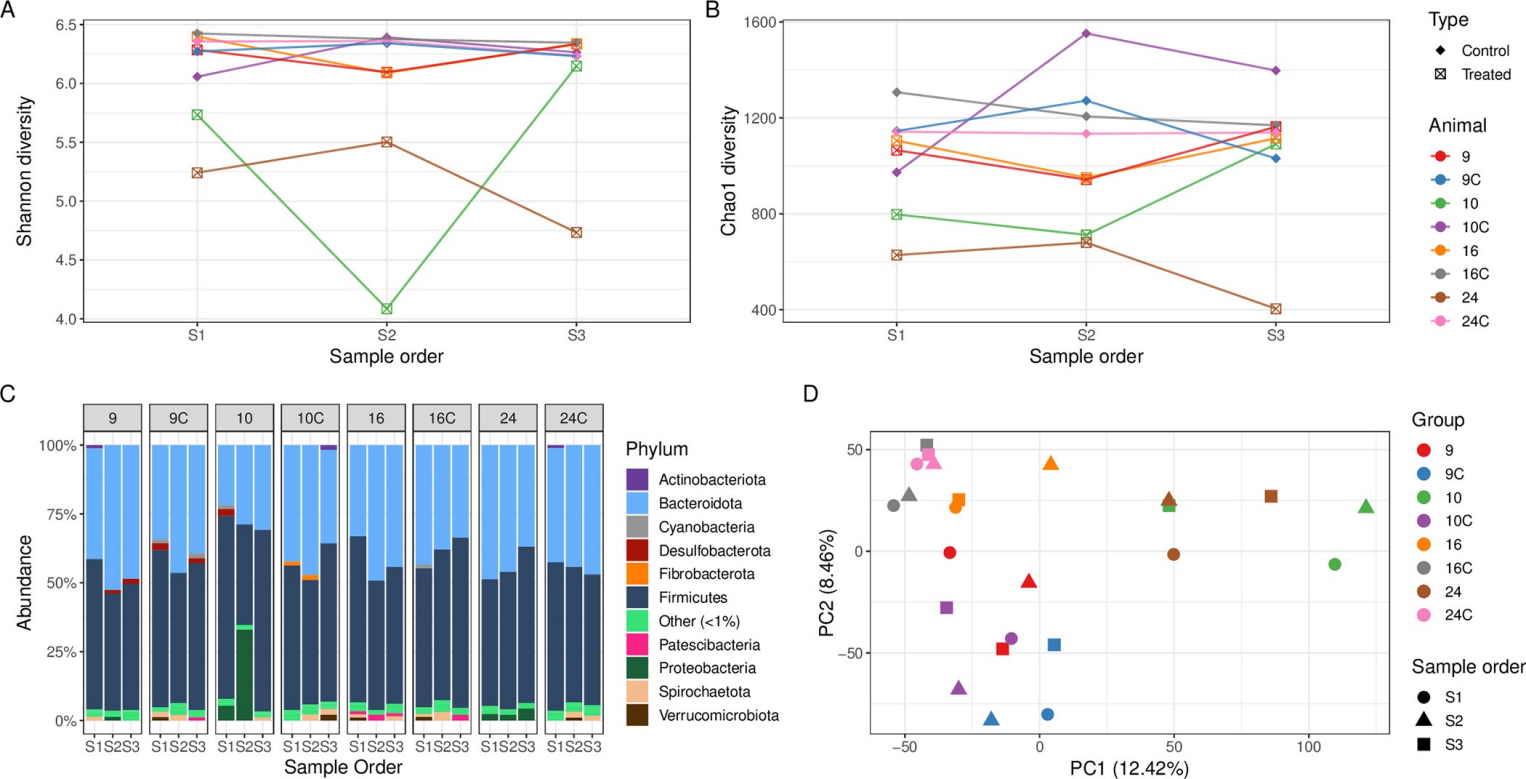
Procaine penicillin G case study. Shannon (**A**) and Chao1 (**B**) α-diversity, and relative abundance of the phyla (**C**) from faecal samples from cows treated with procaine penicillin G (n = 3) and respective control cows (n = 3). Principal Component Analysis (**D**) for the faecal microbiome of treated and control cows at the individual animal level (colours) over the sampling period (shapes). The percentage of variation explained in the Principal Component Analysis is indicated on the axis labels.

Compared to the other treated and control cows, cow 24 (retained foetal membrane) had a lower Shannon and Chao1 α-diversity across all faecal samples collected (S1, S2, S3), except for S2 collected from cow 10. For cow 10 (displaced abomasum), compared to the pre- and post-treatment faecal samples, the Shannon and Chao1 α-diversity was lower for sample S2 (Fig 4A and 4B). The faecal samples from treated cows 9 (foot rot) and 16 (swollen vulva) had a similar Shannon α-diversity compared to the control cows, however, the Chao1 α-diversity was lower for the S2 samples from all four treated cows, suggesting that there were fewer unique ASVs from these faecal samples (Fig 4A and 4B). The Chao1 α-diversity of S3 samples from cows 9, 10, and 16 was similar compared to the control cows (S1, S2 and S3), whereas the S3 Chao1 α-diversity for cow 24 was the lowest across all faecal microbiomes in this case study.

At the phylum level, Firmicutes, which predominantly consists of Gram-positive bacteria, decreased during treatment for cows 9, 10 and 16, although control cows 9C and 10C also showed a decrease in the relative abundance of Firmicutes in sample S2 (Fig 4C). The relative abundance of Proteobacteria increased during treatment (S2) for cow 10 but was not detected post-treatment (Fig 4C). For cow 10, *Escherichia*-*Shigella* were present in S1 (3.0%), with a higher relative abundance in S2 (30.7%) and were in low abundance in S3 (0.01%) post-treatment (S2 Table). The relative abundance of *Escherichia-Shigella* also increased post-treatment for cow 24 (S3, 2.8%), compared to pre- (S1, 0.03%) and during treatment (S2, 0.02%) samples.

The faecal microbiomes from the control cows were more clustered in the PCA plot (Fig 4D) compared to the cows treated with procaine penicillin G (n = 4). The faecal microbiomes of treated cows 10 and 24 were more dispersed on the PCA plot compared to cows 16 and 9, which is consistent with the perturbations in the faecal microbiome observed at the genus level. The faecal microbiomes from individual animals (S1, S2 and S3) generally clustered together, suggesting that microbiome variation is driven at the individual animal level.

### Penethamate hydriodide treated cows

Three cows were treated for mastitis with penethamate hydriodide during the study period (S1 Table). These mastitis cases were diagnosed by positive culture and/or had culture susceptibility testing undertaken prior to treatment, highlighting the prudent antimicrobial stewardship on these two farms. One cow was used as the untreated control (18C/19C) for two treated cows as they started antimicrobial treatment started on the same day.

**Fig 5:**
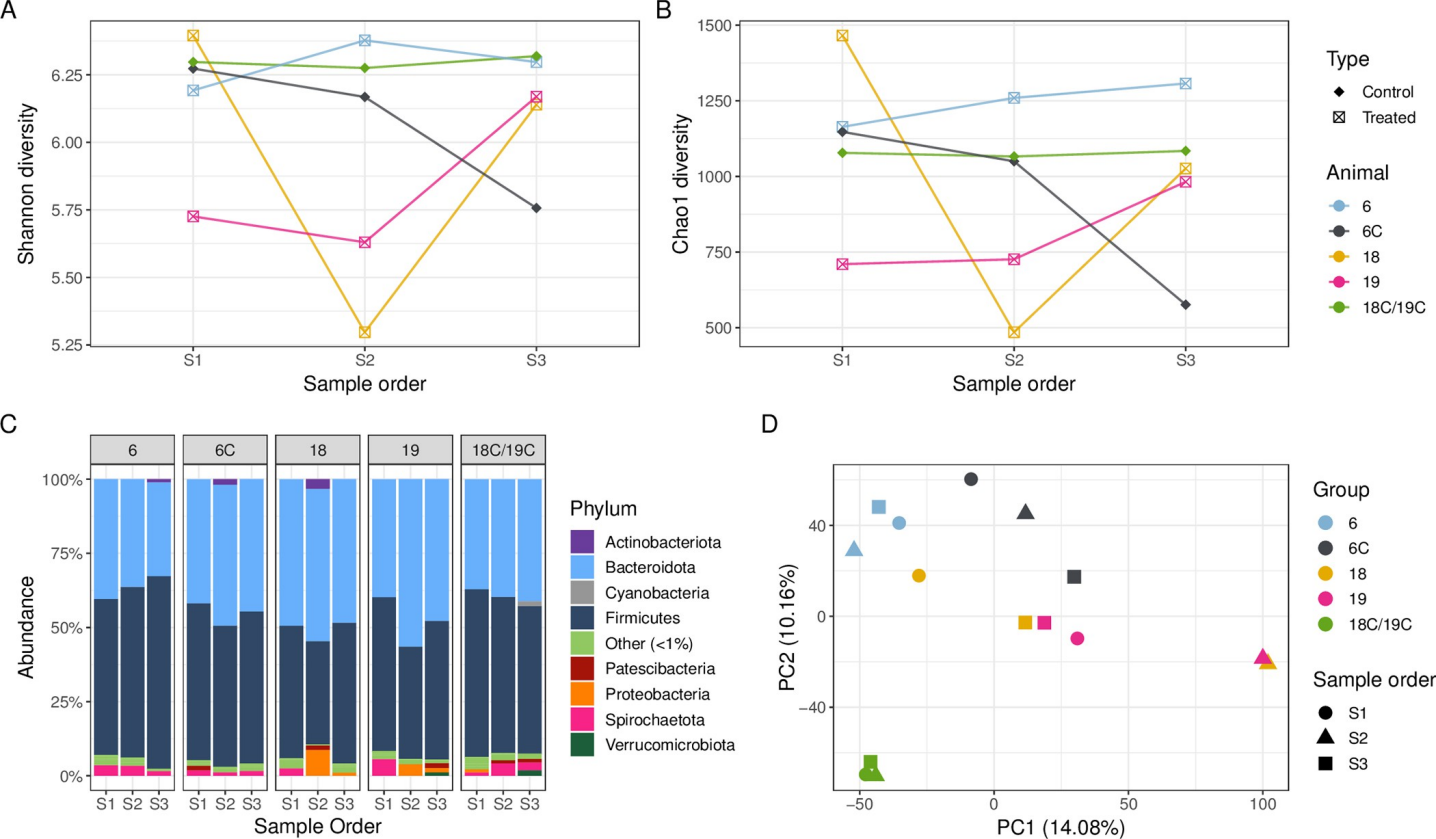
Penethamate hydriodide case study. Shannon (**A**) and Chao1 (**B**) α-diversity, and relative abundance of the phyla (**C**) from faecal samples from cows treated with penethamate hydriodide (n = 3) and respective control cows (n = 3). Principal Component Analysis (**D**) for the faecal microbiome of treated and control cows at the individual animal level (colours) over the sampling period (shapes). The percentage of variation explained in the Principal Component Analysis is indicated on the axis labels.

The Shannon diversity was comparatively lower from the pre-treatment and during treatment samples (S1 and S2) from cow 19 and the S2 sample from cow 18 (Fig 5A). The α-diversity of post-treatment samples (S3) from cows 18 and 19 were more similar to control cows compared to the pre- and during-treatment samples. In comparison, both the Shannon and Chao1 diversity of all three faecal samples from treated cow 6 (S1, S2, S3) were similar to the α-diversity observed in control cows (Fig 5A and 5B), whereas sample S3 from control cow 6C showed a decrease in both the Shannon and Chao1 α-diversity.

For cows 18 and 19, the relative abundance of Proteobacteria increased during treatment (S2) compared to pre-treatment (Fig 5C) with *Escherichia*-*Shigella* having a higher relative abundance in S2 (5.9% and 2.6%) compared to samples S1 (0.01% and 0.03%) and S3 (0% each) for both cows, respectively (S2 Table). *Escherichia-Shigella* were present in low abundance in the faecal samples from treated cow 6 and the control cows (S2 Table). This increase in abundance of *Escherichia-Shigella* during treatment (S2) with penethamate hydriodide for cows 18 and 19 may have been due to less competition with Gram-positive bacteria. The faecal microbiome samples clustered at the individual animal level for control cows 6C and 18C/19C and treated cow 6 (Fig 5D). In comparison, pre- (S1) and post-treatment (S3) samples for cows 18 and 19 were clustered together, however, the during treatment samples (S2) were distinct but clustered together (Fig 5D).

### Marbofloxacin/penethamate hydriodide treated cow

**Fig 6:**
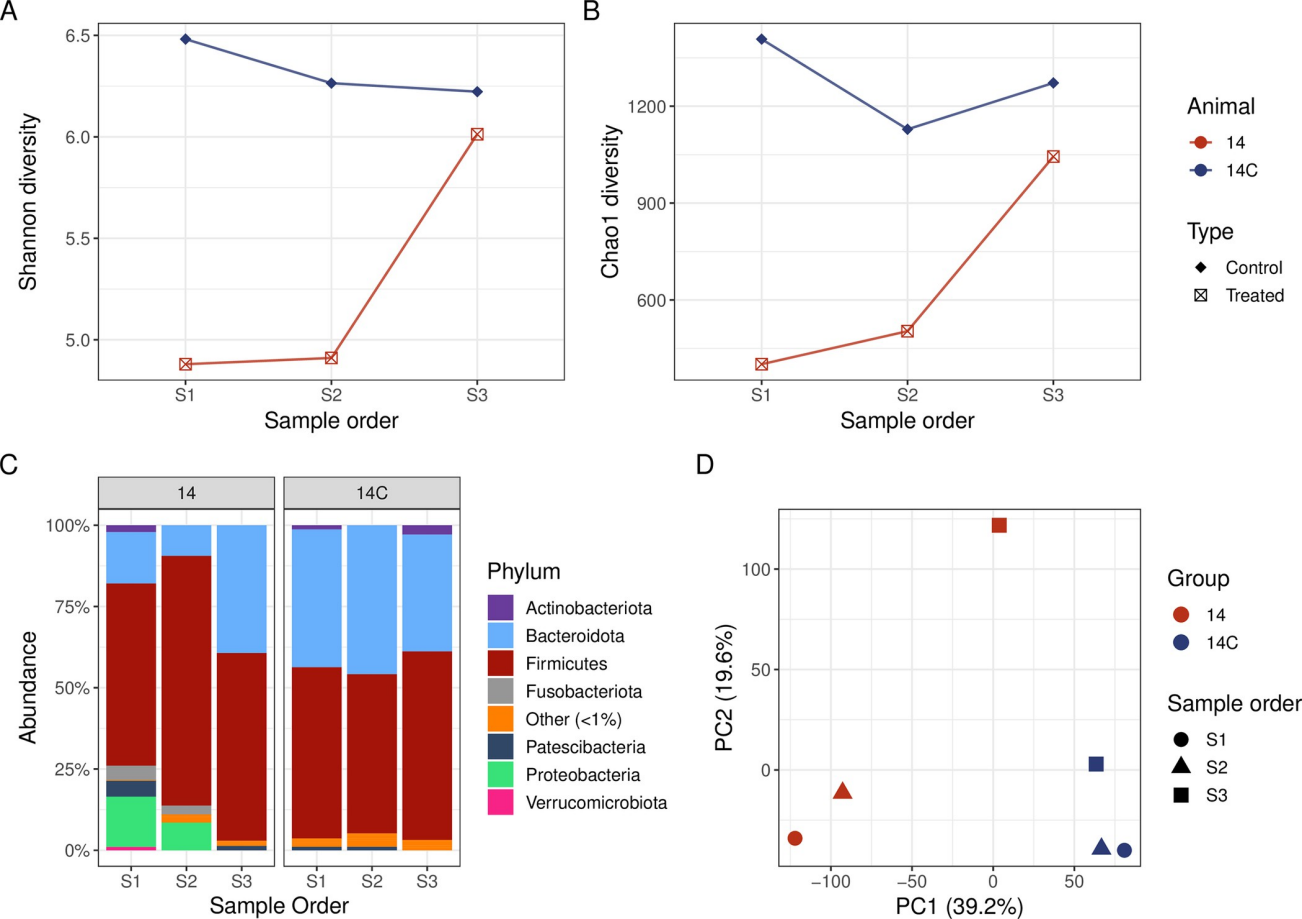
Marbofloxacin/penethamate hydriodide case study. Shannon (A) and Chao1 (B) α-diversity of faecal samples from a cow treated with marbofloxacin/penethamate hydriodide (n = 1) and the respective control cow (n = 1). (C) Relative abundance of the phyla from faecal samples from a cow treated with marbofloxacin/penethamate hydriodide (n = 1) and the respective control cow (n = 1). Principal Component Analysis (D) for the faecal microbiome of the treated and control cow at the individual animal level (colours) over the sampling period (shapes). The percentage of variation explained in the Principal Component Analysis is indicated on the axis labels.

One cow (cow 14) was treated for mastitis with marbofloxacin and penethamate hydriodide and was treated with the intramammary antibiotic Mastalone® (oxytetracycline, oleandomycin, and neomycin) 24 hours before the pre-treatment sample was collected. The Shannon and Chao1 α-diversity of the faecal microbiome of the untreated control cow in this case study (14C) was relatively high and consistent across all sample periods (S1, S2 and S3), which indicates a high level of bacterial diversity (Fig 6A and 6B). In comparison, pre (S1) and during treatment (S2) samples collected from treated cow 14 had a comparatively lower Shannon and Chao1 diversity compared to the post-treatment sample (S3), which is likely due to the antibiotic treatment. The α-diversity of sample S3 increased post-treatment and was more similar to the diversity observed in the control cow faecal microbiomes than to S1 or S2 from treated cow 14.

At the phylum level, minor perturbations were observed over the sampling period for control cow 14C (Fig 6C). In comparison, the faecal microbiome of treated cow 14 varied during the sampling period, with an increase in the relative abundance of Firmicutes and a decrease in Bacteroidota during treatment (S2; Fig 6C). The relative abundance of Proteobacteria was also higher in pre- (S1) and during treatment (S2) samples compared to post-treatment (S3). The faecal microbiome samples clustered at the individual animal level for control cow 14C, whereas for treated cow 14 the pre- (S1) and during treatment (S2) samples were clustered together and the post-treatment sample was distinct (Fig 6D).

For treated cow 14, at the genus level the microbiome composition of S1 was distinct, with genera detected that were absent or in low abundance in S2 and S3 and the control cow 14C (S1, S2 and S3) such as *Burkholderia*/*Caballeronia*/*Paraburkholderia* (these could not be differentiated at a higher taxonomic resolution), *Clostridium* sensu stricto 1, *Succiniclasticum*, and *Terrisporobacter* (S2 Table). In cow 14, the microbiome composition was altered during treatment (S2), with a higher relative abundance of the genera *Lachnoclostridium* (3.0%), *Prevotellaceae* UCG−001 (4.6%) and UCG-005 (29.8%) detected compared to S1 (0.2%, 0.8%, 1.5%, respectively) and S3 (0%, 0%, 12.8%, respectively) samples. For treated cow 14, *Prevotellaceae* UCG-003 were detected in faecal sample S1 (3.8%) and the relative abundance of this genera decreased during antimicrobial treatment (S2, 0.8%) and flourished post-treatment (S3, 12.1%). *Prevotellaceae* UCG-003 was detected at a similar relative abundance in all control cow faecal samples (S1, 7.6%; S2, 9.6%; S3, 9.0%). *Escherichia*-*Shigella* were more abundant in S1 (10.7%) and S2 (7.9%) for treated cow 14 but were not detected in S3 from cow 14 and were in low relative abundance in the control cow faecal microbiome (S1, 0.01%; S2, 0.04%; S3, 0%). The genera detected in the post-treatment sample (S3) for cow 14 were more similar to the faecal microbiome of the control cow (14C) compared to the pre- and during-treatment samples for cow 14.

### Ceftiofur treated cows

**Fig 7:**
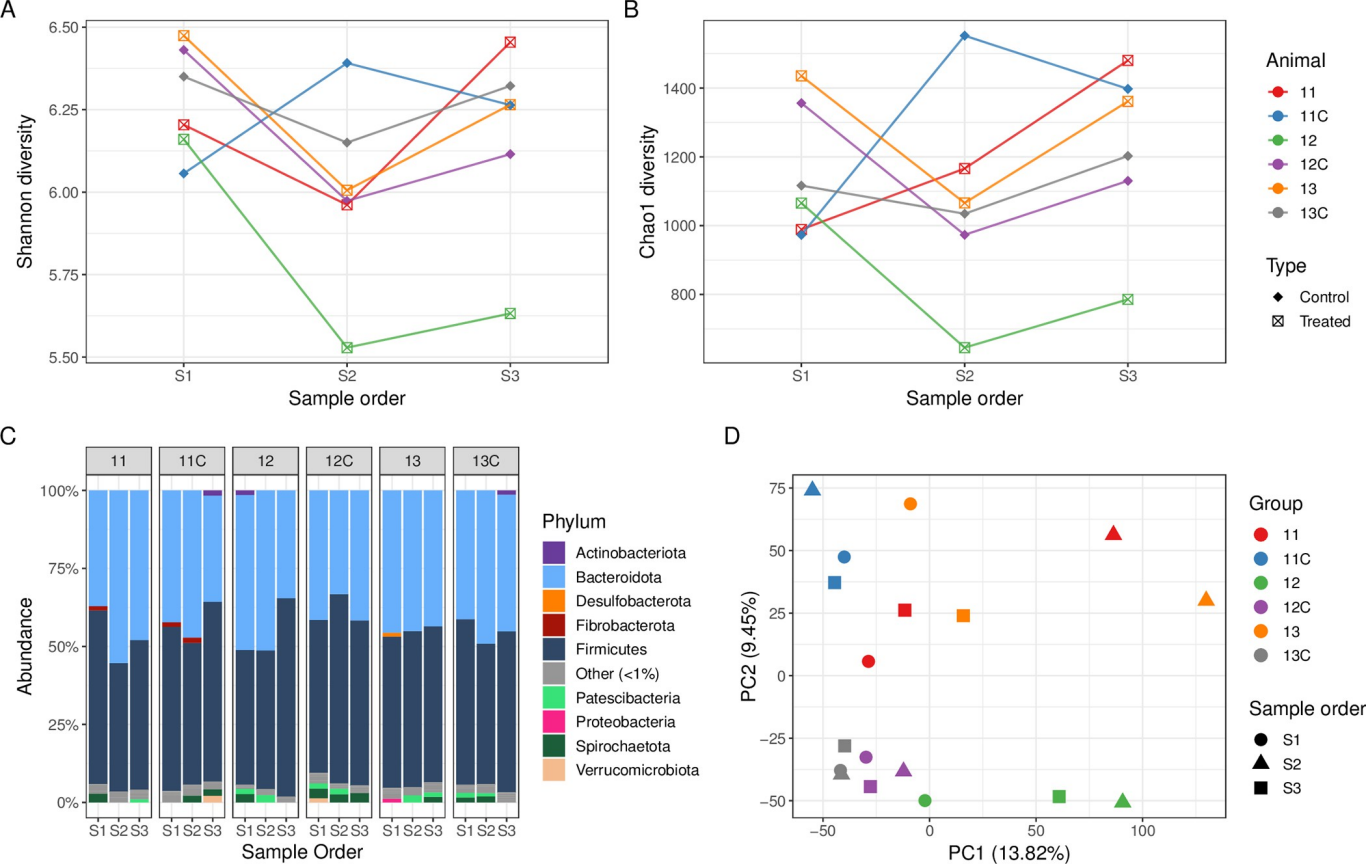
Ceftiofur case study. Shannon (A) and Chao1 (B) α-diversity, and relative abundance of the phyla (C) from faecal samples from cows treated with ceftiofur (n = 3) and respective control cows (n = 3). Principal Component Analysis (D) for the faecal microbiome of treated and control cows at the individual animal level (colours) over the sampling period (shapes). The percentage of variation explained in the Principal Component Analysis is indicated on the axis labels.

Three cows were treated with ceftiofur during the study period (S1 Table). Both the Shannon and Chao1 α-diversity was similar across samples S1 for both treated and control cows (Fig 7A and 7B). The α-diversity remained relatively consistent across the treated and control cows, except for that of sample S2 from control cow 11C where the α-diversity increased and samples S2 and S3 from treated cow 12 where the α-diversity was reduced during and post-treatment compared to pre-treatment (S1) levels.

For cow 13, Proteobacteria were present in a low abundance pre-treatment (S1) and were not detected during (S2) and post-treatment (S3) (Fig 7C). According to PCA analysis, the three faecal microbiomes (S1, S2, and S3) from individual control cows were clustered together (cows 11C, 12C and 13C). However, although samples S2 and S3 from cow 12 clustered together, the S2 samples for treated cows 11 and 13 were distinct (Fig 7D).

Differential abundance analysis was performed at the genus level between treated (n = 3) and control cows (n = 3) in the ceftiofur case study. At the genus level, no differentially abundant genera (p-value >0.05) were identified between pre-treatment samples (S1) collected from treated and control cows. Differential abundance analysis identified significant differences (p-value <0.05) in genera between during treatment (S2) faecal samples from treated (n = 3) and control cows (n = 3), with 14 genera more highly abundant and 14 genera less abundant in treated cows (S3 Table). The differentially abundant genera predominantly belonged to the phylum Firmicutes (19 of 28 genera; 67.9%). For post-treatment (S3) faecal samples, fewer differentially abundant genera were identified compared to during treatment (S2) samples, with one genus less abundant in post-treatment (S3) samples from treated cows compared to the respective control samples (S3 Table). Among the faecal samples collected from treated cows (n = 3), compared to pre-treatment (S1) faecal samples, six genera were more highly abundant, and three genera were less abundant during treatment (S2). Similarly, five genera were identified in higher abundance and nine in lower abundance during treatment (S2) compared to post-treatment (S3). No differentially abundant genera (p-value >0.05) were identified in pre-treatment (S1) compared to post-treatment (S3) samples for treated cows nor between any control cow faecal samples (S1, S2 and S3).

### The emergence of antibiotic resistant *E*. *coli*

The presence and potential emergence of third-generation cephalosporin-resistant *E*. *coli* was investigated in DNA extracted from faecal samples (S1, S2 and S3) obtained from treated and control animals, corresponding culture enrichments, and from resistant *E*. *coli* recovered from selective agar plates. Using PCR, faecal DNA extractions and boiled lysates from the corresponding enrichments from two faecal samples (DG079 (S2) and DG082 (S3)) from cow 12 generated amplicons of the expected size with the CITM primer set [34], indicative of a LAT-1 to LAT-4, CMY-2 to CMY-7 or BIL-1 type β-lactamase. Faecal DNA and enrichments from the remaining ceftiofur treated cows (n = 2) or associated control cows (n = 3) however, were all PCR-negative. Presumptive third-generation cephalosporin-resistant *E*. *coli* were isolated on selective agar plates onto which enrichments from faecal samples S2 and S3 collected from the treated cow 12 (Table 1) were plated. No *E*. *coli* were isolated from the faecal enrichment cultures inoculated onto selective agar plates for sample S1 from cow 12 nor from the faecal samples of other ceftiofur treated or control cows.

**Table 1:** Isolates identified from faecal enrichments of ceftiofur treated cows (n = 3) and associated control cows (n = 3).

Animal	Lab ID	Farm	Type	Sample order^a^	Isolates (n)	MALDI-TOF ID
11	DG067	Dairy 4	Treated	S1	0	-
11	DG073	Dairy 4	Treated	S2	0	-
11	DG087	Dairy 4	Treated	S3	0	-
11C	DG069	Dairy 4	Control	S1	0	-
11C	DG074	Dairy 4	Control	S2	0	-
11C	DG088	Dairy 4	Control	S3	0	-
12	DG070	Dairy 1	Treated	S1	0	-
12	DG079	Dairy 1	Treated	S2	6	*E*. *coli*
12	DG082	Dairy 1	Treated	S3	4	*E*. *coli*
12C	DG071	Dairy 1	Control	S1	4	*Morganella morganii*
12C	DG080	Dairy 1	Control	S2	0	-
12C	DG083	Dairy 1	Control	S3	0	-
13	DG075	Dairy 4	Treated	S1	0	-
13	DG077	Dairy 4	Treated	S2	0	-
13	DG084	Dairy 4	Treated	S3	0	-
13C	DG076	Dairy 4	Control	S1	0	-
13C	DG078	Dairy 4	Control	S2	0	-
13C	DG085	Dairy 4	Control	S3	0	-

^a^ Faecal samples were collected pre- (S1), during (S2) and post-treatment (S3) from dairy cows undergoing a caesarean. Healthy control (untreated) cow samples were collected during the same sampling period.

*E*. *coli* isolated from selective agar plates (n = 10) from faecal enrichments DG079 (S2) and DG082 (S3) from cow 12 were phenotypically resistant to cefotaxime, cefpodoxime and cefoxitin and susceptible to tetracycline, streptomycin and ciprofloxacin, except for isolate DG079e which showed intermediate resistance to cefoxitin. According to phenotypic testing, all isolates were AmpC positive and ESBL negative. Using PCR, all *E*. *coli* (n = 10) were positive for the CITM primer set [34], indicative of a LAT-1 to LAT-4, CMY-2 to CMY-7 or BIL-1 type β-lactamase.

### Whole genome sequencing

The three *E*. *coli* isolates were the same serotype (O160:H32) and sequence type (ST5514) and were genetically similar (Table 2), differing by 53 to 170 core genome SNPs (Table 3). Isolates DG079c and DG082f (isolated from S2 and S3, respectively) were the most similar, differing by only 53 SNPs.

**Table 2:** Genome characteristics of three *E*. *coli* isolates.

Isolate	Contigs	Genome size (bp)	GC (%)	Read depth	N50 (bp)	Sequence type	Serotype
DG079c	112	4,880,186	50.3	120x	110,495	5514	O160:H32
DG079h	308	4,789,972	50.3	82x	37,402	5514	O160:H32
DG082f	128	4,875,060	50.8	89x	91,090	5514	O160:H32

**Table 3:** Core genome single nucleotide polymorphism analysis of three AmpC-producing *E*. *coli* from this study.

Isolate	DF0049.2e	DG079c	DG079h	DG082f
DF0049.2e (reference)	0	61,753	59,783	62,032
DG079c	61,753	0	153	53
DG079h	59,783	153	0	170
DG082f	62,032	53	170	0

The antimicrobial resistance genes *bla*_CMY-2_, chromosomal *ampC*, *aph(6)-Id*, *dfrA14* and *sul2*, potentially conferring resistance to the β-lactam, aminoglycoside, trimethoprim, and sulfonamide classes, respectively were detected in all three *E*. *coli* genomes. The detection of the *bla*_CMY-2_ gene was consistent with resistance to the third-generation cephalosporins. In contrast, all three isolates carried the *aph(6)-Id* gene which confers resistance to streptomycin [57], yet the *E*. *coli* were susceptible to streptomycin using antimicrobial susceptibility testing. Phenotypic testing for trimethoprim and sulfonamide resistance was not undertaken. MOB-suite [53, 54] was used to reconstruct and type the plasmids identified in the *E*. *coli* isolates, with four plasmids identified in DG079c and DG079h and three plasmids detected in DG082f (S4 Table). All three isolates carried an IncI-Ƴ/K1 plasmid encoding the *bla*_CMY-2_ gene and *cia* (Colicin Ia), and a smaller plasmid (8,456 bp) encoding the antimicrobial resistance genes *aph(6)-Id*, *aph(3”)-Ib*, *sul2* and *dfrA14* (S4 Table). However, as these are draft genomes, the plasmids are incomplete and additional long-read sequencing to close the plasmid nucleotide sequence is required to fully characterise the plasmids. The isolates are genetically similar (Tables 2 and 3) and isolated from the same animal over a short time, which further suggests that the detected variation in plasmid characteristics may be due to the limitations of short-read sequencing and the lack of complete plasmid sequences. All isolates harboured seven virulence factors (*astA*, *cia*, *f17A*, *f17G*, *iss*, *terC*, *traT*), and DG079h also carried the *gad* gene (glutamate decarboxylase). No Shiga toxin-producing *E*. *coli* or enteropathogenic *E*. *coli* associated virulence genes were identified among the three *E*. *coli* isolates.

## Discussion

Although several previous studies have demonstrated a variety of impacts of antibiotic therapy (including antibiotic administration as a feed additive) on the faecal microbiomes of healthy cattle [4, 7, 58, 59], none have investigated microbiome perturbations from clinically diseased cows, through antibiotic therapy and post-antibiotic treatment. In this study, undertaken on two NZ working farms, it was hypothesised that clinical disease and the associated systemic antimicrobial treatment would impact the faecal microbiome by decreasing species diversity and reducing the abundance of susceptible bacterial populations, allowing non-susceptible bacterial populations to flourish.

The pre-treatment sample (S1) enabled analysis of the bovine faecal microbiome impacted by a number of clinical diseases of contrasting severity, prior to antimicrobial treatment. There was no overall significant decrease of bacterial diversity and richness prior to antibiotic treatment (S1) in diseased animals compared to controls, however there were several outlier data points from some diseased animals (Fig 2) which corresponded to clinical conditions such as retained foetal membranes/metritis, displaced abomasum, and mastitis suggesting that the large bowel microbiome was already affected by ongoing pathology prior to treatment. The results of this study demonstrate that systemic antimicrobial treatment with either procaine penicillin G, ceftiofur, penethamate hydriodide, or marbofloxacin/penethamate hydriodide reduced the bacterial diversity and richness during treatment (S2) in the bovine faecal microbiome (Figs 4–7, panels A and B), which was likely a result of the antimicrobials targeting susceptible bacteria. Perturbations in the bovine faecal microbiome were more pronounced in cows with severe disease (e.g., left displacement of the abomasum compared to foot rot), but generally, the α-diversity recovered post-antimicrobial treatment (S3) increasing when the cow re-entered the milking herd. Only one faecal sample was collected for each sampling period and the collection of additional samples more frequently during and post-treatment may have enabled a deeper analysis of the microbiome perturbations and recovery post systemic antimicrobial treatment.

Other studies have also demonstrated a reduction in the bovine faecal microbiome richness post antimicrobial treatment [7, 12, 60]. In comparison, the difference in α-diversity between cattle fed a diet supplemented with tylosin compared to control cattle at feedlots in the USA was not statistically significant [61], although treated cattle had a lower α-diversity compared to that of controls. No significant difference in microbiome diversity or richness was observed between healthy beef cattle treated with tulathromycin (n = 15) or controls (n = 15) at a commercial feedlot [58]. These data highlight that numerous factors can affect the microbiome richness and diversity, such as the spectrum of antimicrobial activity as well as host-dependent factors.

Much of the divergence in microbiomes is due to variability at the individual animal level; our study includes additional external factors being set in a natural farm environment, with variation in animal health status, prescribed product, and treatment duration in individual animals as well as temporal changes in seasonality and feed type at the farm level. Many external factors (e.g. feed, weather conditions) were controlled for by sampling sick cows and healthy controls from the same farm over the same time period. However, only a small number of sick cows were eligible for inclusion in this study, which may be partially due to the prudent antimicrobial stewardship on the two research farms. The two research farms also used a low proportion of antimicrobials classified as red tier by the NZVA during the study period. To account for such variation, alterations in the bovine faecal microbiome were analysed at the individual animal level and compared with control animals. The treated and control cows had access to the same feed sources on farm, therefore, food composition is unlikely to be responsible for microbiome differences. However, antimicrobial therapy, immune response, and behavioural changes such as any altered feed intake of diseased cattle may have an impact on the microbiome. Previous studies have shown that environmental factors can impact the bovine faecal microbiome composition, such as transport to the feedlot, time post antimicrobial treatment [7] and geographic location [59]. Therefore, environmental and farm management factors can also have a significant effect on the bovine faecal microbiome.

The spectrum of antimicrobial activity can impact perturbations in the bacterial community composition and specific examples of a reduction in potentially susceptible bacteria can be seen in individual animals. *In vitro* procaine penicillin G has activity against Gram-positive and Gram-negative bacteria, however, it is conventionally used to treat infections caused by susceptible Gram-positive bacteria such as *Streptococcus* spp. [62]. Within the procaine penicillin G case study (Fig 4), treated cow 10 had an increase in the relative abundance of *Escherichia*-*Shigella* pre- and during treatment and an increase in *Bacteroidales* during treatment (S3 Table). In contrast, there was an increase in the relative abundance of the Bacteroidota for cow 16 during treatment (Fig 4C); the increase in Gram-negative bacteria during treatment within the faecal microbiomes of these animals may be due to reduced competition with Gram-positive bacteria, which are also generally susceptible to procaine penicillin G.

Penethamate hydriodide (Penethaject) is considered active against Gram-positive bacteria [17, 62]. The increase in the relative abundance of Proteobacteria during treatment for cows 18 and 19 (Fig 5C) may have been due to a reduction in Gram-positive bacteria allowing for Gram-negative bacteria to persist and increase in relative abundance. Marbofloxacin is a third-generation fluoroquinolone developed for veterinary treatment and has a broad spectrum of activity against most Gram-negative and some Gram-positive bacteria. Simultaneous treatment with both marbofloxacin and penethamate hydriodide would likely alter the faecal microbiome during and post-treatment by reducing the abundance of susceptible bacteria, due to the combined wide spectrum of activity of these two antimicrobials. For treated cow 14, the reduction in the relative abundance of Bacteroidota (Fig 6C) may be due to the activity spectrum of marbofloxacin against Gram-negative bacteria. The relative abundance of *Escherichia*-*Shigella* was higher in S1 and S2 for treated cow 14 and decreased post-treatment, supporting this hypothesis.

Ceftiofur has a broad spectrum of activity against a variety of Gram-negative and Gram-positive bacteria. The use of third- and fourth-generation cephalosporins has been reported as a risk factor for the detection of extended-spectrum β-lactamase (ESBL)- and AmpC-producing *E*. *coli* on dairy farms [63], and an increase in β-lactam resistance genes has been detected in healthy dairy cows treated with ceftiofur [4]. Differentially abundant genera between treated (n = 3) and control (n = 3) cows belonged mainly to the phylum Firmicutes (mostly Gram-positive) which is one of the predominant phyla in the faecal samples from this case study (Fig 7C). A comparison of pre-treatment (S1) faecal samples from treated and control cows did not detect any differentially abundant genera (p-value >0.05), which may be due to all the animals being healthy prior to the procedure and feed, weather, and environmental conditions being similar for both the treated and control cows. The highest number of differentially abundant genera was identified in the during treatment samples between treated and control cows, which reflects the microbiome perturbations during systemic antimicrobial therapy. Within the treated group (S1, S2 and S3), the highest number of differentially abundant genera were identified during treatment (S2 and S3 Table), highlighting the impact of antibiotic treatment on the bacterial community composition.

Prior to ceftiofur treatment (S1), the phylum Proteobacteria was only detected in one treated cow (13) but was absent during (S2) and post-treatment (S3) (Fig 7C). However, in one treated cow (cow 12) PCR data indicated that a LAT-1 to LAT-4, CMY-2 to CMY-7 or BIL-1 type β-lactamase was detected from DNA extracted from both faeces and faecal culture enrichments. Consequently, AmpC-producing *E*. *coli* were isolated during treatment (48 hours after ceftiofur treatment commenced) and post-treatment (4 days after treatment was completed) from this cow. Molecular characterisation confirmed that the AmpC-producing *E*. *coli* harboured the plasmid-mediated *bla*_CMY-2_ type gene, which is the most prevalent pAmpC gene globally [64]. The absence of the β-lactamase type gene by PCR in both DNA and culture enrichments from the pre-treatment sample (S1) implies that the AmpC-producing *E*. *coli* isolated from the S2 and S3 samples may have been present at levels undetectable with standard methods prior to treatment with third-generation cephalosporin or may have colonised cow 12 and become detectable between the S1 and S2 faecal sampling occasions. The post-treatment sample (S3) was collected when the cow re-entered the dairy herd, and therefore, suggests a potential transmission route for the dissemination of AmpC-producing *E*. *coli* within the dairy herd. Additional samples were not collected post-treatment, therefore the persistence and duration of shedding of the emergent AmpC-producing *E*. *coli* in the faeces of cow 12 is unknown.

An increase in *E*. *coli* resistant to third-generation cephalosporins in cattle faeces has been identified during and post-treatment with ceftiofur in previous culture-based studies. Cows treated with two doses of ceftiofur on USA dairy farms had a higher number of *E*. *coli* resistant to third-generation cephalosporins in the treated group compared to the untreated group by day six, which decreased by days 28 and 56 and was comparable with the untreated group [65]. A higher abundance of *E*. *coli* resistant to third-generation cephalosporins was identified in older milking cows, which may be due to pre-exposure to antimicrobials [65]. *E*. *coli* with reduced susceptibility to ceftriaxone (third-generation cephalosporin) were more likely to be isolated from farms that reported using ceftiofur in the USA, however no associations were identified at the individual animal level in cows treated with ceftiofur within six months of sampling [66]. In contrast, no association was identified between ceftiofur use and cephalosporin resistance in *E*. *coli* across 42 farms across the USA [67], but only ceftiofur use on farm was examined, and the total antimicrobial usage and other antimicrobials were not included in the analysis.

Similar to this study, faecal samples from ceftiofur treated (n = 5; treated for *Leptospira*-associated infertility) and untreated (n = 5) cattle housed in a barn on a dairy farm in the USA were analysed for ceftiofur resistant *E*. *coli* [68]. No ceftiofur resistant *E*. *coli* (n = 265) were isolated from faecal samples collected from untreated cows or before treatment for treated cows (days -1 and 0), and were only isolated from faeces (12 of 203) collected during and directly post-treatment from treated cows [68]. The *bla*_CMY-2_ gene was detected in all 12 ceftiofur resistant *E*. *coli*. These findings, along with the results from this study, suggest that treatment with ceftiofur may enrich for AmpC producing *E*. *coli* and these organisms may be excreted post-treatment.

Genome sequencing revealed that the *E*. *coli* (n = 3) carried the *aph(6)-*Id gene, which encodes a streptomycin phosphotransferase [57], yet all ten of the *E*. *coli* were susceptible to streptomycin in phenotypic testing. Studies have shown that APH(6) enzymes are less efficient at inactivating streptomycin [57] and the *aph(6)-*Id gene is often found in combination with other aminoglycoside resistance genes such as *aph*(3") and *ant*(3"), suggesting that additional 3’-phosphotransferase or 3’-adenylyltransferase activity may be required for sufficient streptomycin inactivation in the presence of *aph(6)-*Id [57]. This finding highlights the necessity to confirm a resistance phenotype and that caution should be used when reporting a resistance phenotype predicted from genotypic information.

Isolation of plasmid-mediated AmpC-producing *E*. *coli* post-treatment with ceftiofur is a public and animal health concern due to the health impacts of resistant infections and the potential dissemination of pathogenic bacteria harbouring clinically relevant antimicrobial resistance genes. The lack of detection of AmpC-producing *E*. *coli* pre-treatment, and culture-based isolation and molecular detection from faecal samples and enrichment broths during and post-treatment corroborates the recommendation to reduce the use of critically important antimicrobials such as third- and fourth-generation cephalosporins [69].

## Conclusions

In this study on working dairy farms with typical bovine clinical diseases and investigating treatment case studies, decreased bacterial microbiome diversity and richness during systemic antimicrobial treatment was observed, but it was found that the α-diversity often recovered post-treatment when the recovered cow re-entered the milking herd. Perturbations in the microbiome composition and the ability of microbiomes to recover varied at the individual animal level, highlighting that animal is a key driver of variation. Additional factors such as disease severity, antimicrobial treatment and duration, and changes in farm management factors are also likely to impact the bovine faecal microbiome. Emergent AmpC-producing *E*. *coli* were isolated from faecal enrichments collected during and post treatment with ceftiofur from one cow. This finding represents a noteworthy case of "AMR in action" and has implications for the development and dissemination of clinically relevant antimicrobial resistance genes as well as supporting the need to limit the use of critically important antimicrobials to reduce the development and spread of AMR.

## Supporting information

S1 Fig Relative abundance of the phyla from faecal samples from cows treated with procaine penicillin G (n = 23). (JPEG)

S1 Table Metadata for faecal samples included in this study. (XLSX)

S2 Table Relative abundance (%) of selected genera from the procaine penicillin G, penethamate hydriodide and marbofloxacin/penethamate hydriodide case studies. (XLSX)

S3 Table Differential abundance analysis at the genus level between treated (n = 3) and control cows (n = 3) in the ceftiofur case study. (XLSX)

S4 Table Characteristics of plasmids from draft *E*. *coli* assemblies. (DOCX)
