# Supplementary figures and images for: Impact of systemic antimicrobial therapy on the faecal microbiome in symptomatic dairy cows
Source: PLoS One. 2024 Jan 5;19(1):e0296290. doi: 10.1371/journal.pone.0296290 (PMC10769045; doi:10.1371/journal.pone.0296290)

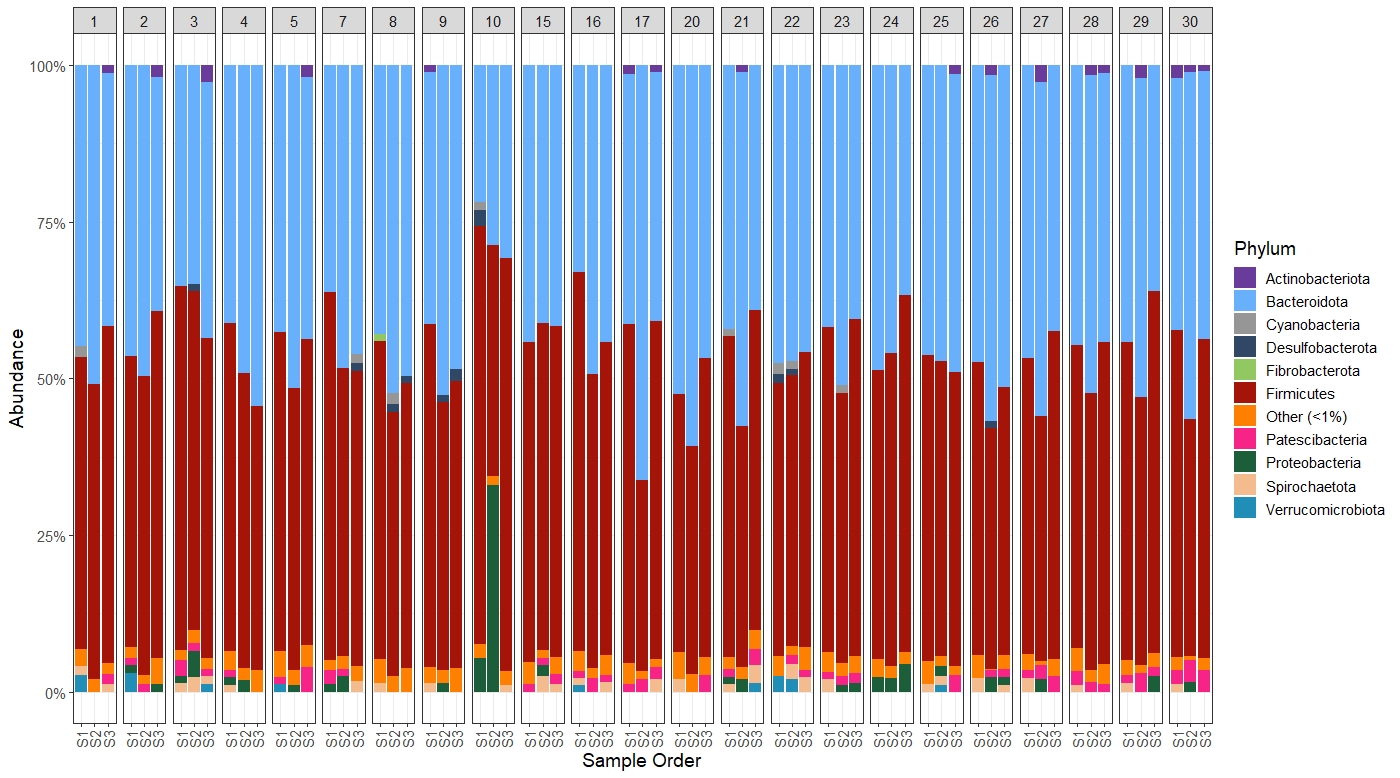

Supplement: S1 Fig — (JPEG) [file pone.0296290.s001.jpeg]
