# Supplementary material for: Impact of systemic antimicrobial therapy on the faecal microbiome in symptomatic dairy cows
Source: PLoS One. 2024 Jan 5;19(1):e0296290. doi: 10.1371/journal.pone.0296290 (PMC10769045; doi:10.1371/journal.pone.0296290)
Supplement: S4 Table — (DOCX) [file pone.0296290.s005.docx]

**Supplementary Table S5:** **Characteristics of plasmids from draft *E. coli* assemblies.**

| **Isolate** | **Plasmid ID** | **No. of contigs** | **Size (bp)** | **GC (%)** | **Replicon type** | **Predicted mobility** | **Taxon name of convergence of literature reported host ranges** | **Antimicrobial resistance genes** | **Virulence genes** | **Mobile genetic elements** |
| --- | --- | --- | --- | --- | --- | --- | --- | --- | --- | --- |
| DG079c | DG079c_1 | 1 | 8,456 | 52.6 | Unknown | Mobilizable | *Enterobacterales* | *aph(6)-Id, aph(3”)-Ib, sul2, dfrA14* | None | IS91 |
| DG079c | DG079c_2 | 6 | 87,590 | 49.9 | IncI-Ƴ/K1 | Conjugative | *Enterobacterales* | *bla*_CMY-2_ | *cia* | None detected |
| DG079c | DG079c_3 | 4 | 56,489 | 51.1 | Unknown | Conjugative | Unknown | None | *traJ, traT* | IS66 |
| DG079c | DG079c_4 | 3 | 19,866 | 50.4 | IncFIB | Non-mobilizable | *Enterobacterales* | None | *f17ACDG* | IS4 |
| DG079h | DG079h_1 | 1 | 8,456 | 52.6 | Unknown | Mobilizable | *Enterobacterales* | *aph(6)-Id, aph(3”)-Ib, sul2, dfrA14* | None | IS91 |
| DG079h | DG079h_2 | 9 | 18,344 | 49.0 | IncFIB | Non-mobilizable | *Enterobacterales* | None | *f17ACDG* | IS30 |
| DG079h | DG079h_3 | 3 | 87,602 | 50.3 | IncI-Ƴ/K1 | Conjugative | *Enterobacterales* | *bla*_CMY-2_ | *cia* | None detected |
| DG079h | DG079h_4 | 1 | 6,681 | 38.7 | Unknown | Non-mobilizable | Unknown | None | None | None detected |
| DG082f | DG082f_1 | 1 | 8,456 | 52.6 | Unknown | Mobilizable | *Enterobacterales* | *aph(6)-Id, aph(3”)-Ib, sul2, dfrA14* | None | IS91 |
| DG082f | DG082f_2 | 6 | 90,287 | 50.7 | IncI-Ƴ/K1 | Conjugative | *Enterobacterales* | *bla*_CMY-2_ | *cia* | None detected |
| DG082f | DG082f_3 | 8 | 65,462 | 51.2 | IncFIB | Conjugative | *Enterobacterales* | None | *f17ACDG, traJ, traT* | IS4 |
